# Diethyl 3*H*-naphtho[2,1-*b*]pyran-2,3-dicarboxyl­ate

**DOI:** 10.1107/S1600536809008691

**Published:** 2009-03-14

**Authors:** Abdullah Mohamed Asiri, Seik Weng Ng

**Affiliations:** a Chemistry Department, Faculty of Science, King Abdul Aziz University, Jeddah, Saudi Arabia; b Department of Chemistry, University of Malaya, 50603 Kuala Lumpur, Malaysia

## Abstract

The *sp* ^3^-hybridized methine C atom in the title compound, C_19_H_18_O_5_, lies out of the mean plane of the remaining 13 atoms of the naphthopyran fused-ring system by 0.571 (1) Å, and its H atom occupies a pseudo-equatorial site.

## Related literature

For a review on 2*H*-naphthopyrans, see: Crano & Guglielmetti (1999 ▶). For the structure of the dimethyl ester analog, see: Ramazani *et al.* (2002 ▶).
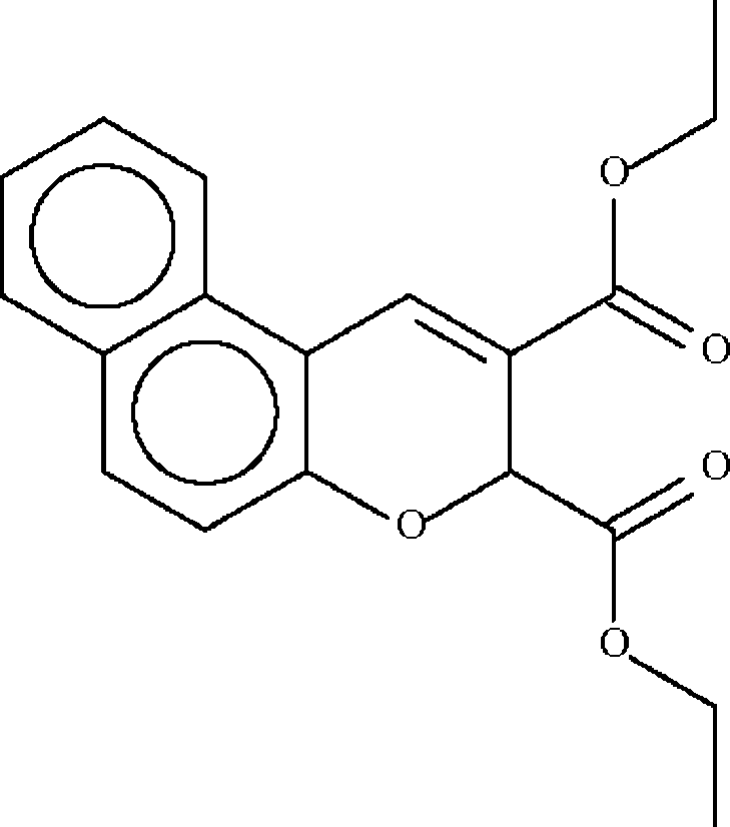


## Experimental

### 

#### Crystal data

C_19_H_18_O_5_
*M* *_r_* = 326.33
Monoclinic, 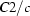

*a* = 28.5156 (3) Å
*b* = 7.5804 (1) Å
*c* = 18.5365 (2) Å
β = 126.413 (1)°
*V* = 3224.54 (6) Å^3^
*Z* = 8
Mo *K*α radiation
μ = 0.10 mm^−1^
*T* = 123 K
0.30 × 0.30 × 0.15 mm

#### Data collection

Bruker SMART APEX diffractometer
Absorption correction: none
14849 measured reflections
3698 independent reflections
3453 reflections with *I* > 2σ(*I*)
*R*_int_ = 0.019

#### Refinement

*R*[*F* ^2^ > 2σ(*F* ^2^)] = 0.039
*wR*(*F* ^2^) = 0.108
*S* = 1.04
3698 reflections
219 parameters
H-atom parameters constrained
Δρ_max_ = 0.25 e Å^−3^
Δρ_min_ = −0.29 e Å^−3^

### 

Data collection: *APEX2* (Bruker, 2008 ▶); cell refinement: *SAINT* (Bruker, 2008 ▶); data reduction: *SAINT*; program(s) used to solve structure: *SHELXS97* (Sheldrick, 2008 ▶); program(s) used to refine structure: *SHELXL97* (Sheldrick, 2008 ▶); molecular graphics: *X-SEED* (Barbour, 2001 ▶); software used to prepare material for publication: *publCIF* (Westrip, 2009 ▶).

## Supplementary Material

Crystal structure: contains datablocks global, I. DOI: 10.1107/S1600536809008691/lh2784sup1.cif

Structure factors: contains datablocks I. DOI: 10.1107/S1600536809008691/lh2784Isup2.hkl

Additional supplementary materials:  crystallographic information; 3D view; checkCIF report

## Figures and Tables

**Fig. 1.**
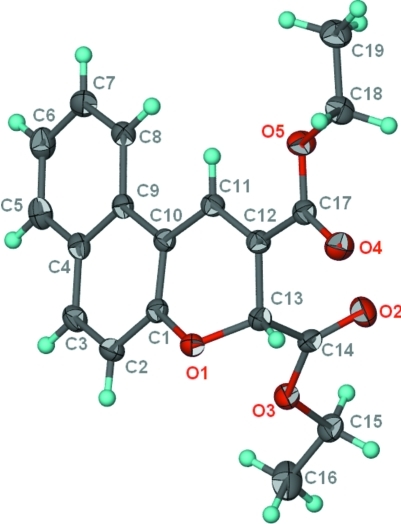
Thermal ellisoid plot (Barbour, 2001) of C19H18O5; probability levels are set at 70% and H-atoms are drawn as spheres of arbitrary radius.

## supplementary crystallographic information

### Comment

The molecular structure of the title compound is shown in Fig. 1.

### Experimental

Triphenylphosphine (13.1 g, 0.05 mol) and 2-hydroxy-1-naphthaldehyde (8.6 g, 0.05 mol) were dissolved in dichloromethane (100 ml). The solution was cooled to 263 K. Diethyl acetylenedicarboxylate (8.5 g, 0.05 mol) dissolved in dichloromethane (20 ml) was added over 20 min. The mixture was then stirred for 2 days. The solvent was removed under reduced pressure and the residue was purified by column chromatography over silica gel; ether-toulene was the eluent. The solvent was removed under reduced pressure and the product was obtained as bright yellow crystals by recrystallization from toulene (14.7 g, 90% yield).

### Refinement

Carbon-bound H-atoms were placed in calculated positions (C–H 0.95-1.00 Å) and were included in the refinement in the riding model approximation, with *U*_eq_(H) fixed at 1.2*U*(C) or 1.5*U*_eq_(C) for methyl H atoms.

### Crystal data

**Table:**

C_19_H_18_O_5_	*F*(000) = 1376
*M**_r_* = 326.33	*D*_x_ = 1.344 Mg m^−^^3^
Monoclinic, *C*2/*c*	Mo *K*α radiation, λ = 0.71073 Å
Hall symbol: -C 2yc	Cell parameters from 9911 reflections
*a* = 28.5156 (3) Å	θ = 2.7–28.7°
*b* = 7.5804 (1) Å	µ = 0.10 mm^−^^1^
*c* = 18.5365 (2) Å	*T* = 123 K
β = 126.413 (1)°	Prism, yellow
*V* = 3224.54 (6) Å^3^	0.30 × 0.30 × 0.15 mm
*Z* = 8	

### Data collection

**Table:**

Bruker SMART APEX diffractometer	3453 reflections with *I* > 2σ(*I*)
Radiation source: fine-focus sealed tube	*R*_int_ = 0.019
graphite	θ_max_ = 27.5°, θ_min_ = 1.8°
ω scans	*h* = −36→36
14849 measured reflections	*k* = −9→9
3698 independent reflections	*l* = −23→24

### Refinement

**Table:**

Refinement on *F*^2^	Primary atom site location: structure-invariant direct methods
Least-squares matrix: full	Secondary atom site location: difference Fourier map
*R*[*F*^2^ > 2σ(*F*^2^)] = 0.039	Hydrogen site location: inferred from neighbouring sites
*wR*(*F*^2^) = 0.108	H-atom parameters constrained
*S* = 1.03	*w* = 1/[σ^2^(*F*_o_^2^) + (0.0623*P*)^2^ + 2.3553*P*] where *P* = (*F*_o_^2^ + 2*F*_c_^2^)/3
3698 reflections	(Δ/σ)_max_ = 0.001
219 parameters	Δρ_max_ = 0.25 e Å^−^^3^
0 restraints	Δρ_min_ = −0.29 e Å^−^^3^

### Special details

**Table:**

Geometry. All e.s.d.'s (except the e.s.d. in the dihedral angle between two l.s. planes) are estimated using the full covariance matrix. The cell e.s.d.'s are taken into account individually in the estimation of e.s.d.'s in distances, angles and torsion angles; correlations between e.s.d.'s in cell parameters are only used when they are defined by crystal symmetry. An approximate (isotropic) treatment of cell e.s.d.'s is used for estimating e.s.d.'s involving l.s. planes.

### Fractional atomic coordinates and isotropic or equivalent isotropic displacement parameters (Å^2^)

**Table:**

	*x*	*y*	*z*	*U*_iso_*/*U*_eq_	
O1	0.62940 (3)	0.55357 (10)	0.45186 (5)	0.01852 (17)	
O2	0.50133 (4)	0.28978 (12)	0.34344 (6)	0.0312 (2)	
O3	0.59191 (3)	0.24695 (10)	0.38235 (5)	0.02188 (18)	
O4	0.46280 (3)	0.74298 (11)	0.29129 (5)	0.02427 (19)	
O5	0.46291 (3)	0.79011 (11)	0.41127 (5)	0.02219 (18)	
C1	0.65404 (4)	0.50737 (13)	0.53927 (7)	0.0168 (2)	
C2	0.70965 (5)	0.42956 (14)	0.58737 (7)	0.0204 (2)	
H2	0.7282	0.4094	0.5592	0.025*	
C3	0.73670 (4)	0.38328 (15)	0.67493 (7)	0.0213 (2)	
H3	0.7734	0.3256	0.7067	0.026*	
C4	0.71080 (4)	0.42003 (14)	0.71938 (7)	0.0185 (2)	
C5	0.73888 (5)	0.37331 (15)	0.81041 (7)	0.0230 (2)	
H5	0.7749	0.3117	0.8419	0.028*	
C6	0.71465 (5)	0.41601 (16)	0.85348 (7)	0.0251 (2)	
H6	0.7339	0.3839	0.9145	0.030*	
C7	0.66120 (5)	0.50753 (16)	0.80743 (7)	0.0232 (2)	
H7	0.6454	0.5407	0.8383	0.028*	
C8	0.63177 (4)	0.54922 (14)	0.71821 (7)	0.0191 (2)	
H8	0.5953	0.6080	0.6876	0.023*	
C9	0.65548 (4)	0.50523 (13)	0.67154 (7)	0.0162 (2)	
C10	0.62585 (4)	0.54314 (13)	0.57807 (7)	0.0159 (2)	
C11	0.56863 (4)	0.62474 (13)	0.52185 (7)	0.0166 (2)	
H11	0.5517	0.6760	0.5481	0.020*	
C12	0.53990 (4)	0.62713 (13)	0.43245 (7)	0.0173 (2)	
C13	0.56717 (4)	0.53746 (14)	0.39323 (7)	0.0180 (2)	
H13	0.5530	0.5992	0.3360	0.022*	
C14	0.54901 (4)	0.34276 (15)	0.37069 (7)	0.0193 (2)	
C15	0.58056 (5)	0.06142 (14)	0.35676 (8)	0.0238 (2)	
H15A	0.5534	0.0495	0.2907	0.029*	
H15B	0.5631	0.0029	0.3834	0.029*	
C16	0.63836 (6)	−0.01934 (18)	0.39174 (10)	0.0373 (3)	
H16A	0.6331	−0.1447	0.3758	0.056*	
H16B	0.6646	−0.0070	0.4571	0.056*	
H16C	0.6552	0.0409	0.3652	0.056*	
C17	0.48480 (4)	0.72331 (13)	0.37016 (7)	0.0178 (2)	
C18	0.41183 (5)	0.90167 (15)	0.35647 (7)	0.0234 (2)	
H18A	0.3810	0.8389	0.3012	0.028*	
H18B	0.4219	1.0111	0.3393	0.028*	
C19	0.39141 (5)	0.94429 (16)	0.41299 (8)	0.0275 (3)	
H19A	0.3586	1.0269	0.3806	0.041*	
H19B	0.4234	0.9981	0.4695	0.041*	
H19C	0.3789	0.8357	0.4257	0.041*	

### Atomic displacement parameters (Å^2^)

**Table:**

	*U*^11^	*U*^22^	*U*^33^	*U*^12^	*U*^13^	*U*^23^
O1	0.0179 (4)	0.0226 (4)	0.0170 (3)	−0.0018 (3)	0.0114 (3)	−0.0014 (3)
O2	0.0211 (4)	0.0299 (5)	0.0412 (5)	−0.0049 (3)	0.0177 (4)	−0.0129 (4)
O3	0.0194 (4)	0.0195 (4)	0.0260 (4)	−0.0008 (3)	0.0130 (3)	−0.0048 (3)
O4	0.0234 (4)	0.0296 (4)	0.0181 (4)	0.0041 (3)	0.0114 (3)	0.0033 (3)
O5	0.0202 (4)	0.0256 (4)	0.0192 (4)	0.0079 (3)	0.0108 (3)	0.0024 (3)
C1	0.0174 (5)	0.0157 (4)	0.0170 (4)	−0.0031 (4)	0.0101 (4)	−0.0024 (4)
C2	0.0180 (5)	0.0216 (5)	0.0246 (5)	−0.0010 (4)	0.0143 (4)	−0.0039 (4)
C3	0.0155 (5)	0.0214 (5)	0.0243 (5)	0.0015 (4)	0.0104 (4)	−0.0006 (4)
C4	0.0161 (5)	0.0169 (5)	0.0202 (5)	−0.0019 (4)	0.0095 (4)	−0.0007 (4)
C5	0.0175 (5)	0.0233 (5)	0.0216 (5)	−0.0006 (4)	0.0079 (4)	0.0034 (4)
C6	0.0226 (5)	0.0308 (6)	0.0169 (5)	−0.0041 (4)	0.0091 (4)	0.0033 (4)
C7	0.0232 (5)	0.0291 (6)	0.0202 (5)	−0.0041 (4)	0.0144 (4)	−0.0010 (4)
C8	0.0178 (5)	0.0211 (5)	0.0195 (5)	−0.0015 (4)	0.0116 (4)	−0.0007 (4)
C9	0.0158 (4)	0.0149 (4)	0.0174 (5)	−0.0025 (3)	0.0095 (4)	−0.0012 (3)
C10	0.0156 (4)	0.0144 (4)	0.0174 (5)	−0.0011 (3)	0.0096 (4)	−0.0014 (3)
C11	0.0175 (5)	0.0151 (4)	0.0190 (5)	−0.0005 (4)	0.0117 (4)	−0.0009 (4)
C12	0.0178 (5)	0.0162 (5)	0.0188 (5)	0.0000 (4)	0.0113 (4)	−0.0005 (4)
C13	0.0165 (5)	0.0212 (5)	0.0152 (4)	0.0003 (4)	0.0088 (4)	−0.0007 (4)
C14	0.0190 (5)	0.0230 (5)	0.0159 (4)	0.0000 (4)	0.0103 (4)	−0.0031 (4)
C15	0.0234 (5)	0.0187 (5)	0.0268 (5)	−0.0021 (4)	0.0135 (5)	−0.0060 (4)
C16	0.0255 (6)	0.0254 (6)	0.0442 (7)	0.0049 (5)	0.0115 (6)	−0.0061 (5)
C17	0.0182 (5)	0.0158 (4)	0.0191 (5)	−0.0013 (4)	0.0109 (4)	−0.0006 (4)
C18	0.0202 (5)	0.0216 (5)	0.0248 (5)	0.0067 (4)	0.0114 (4)	0.0037 (4)
C19	0.0271 (6)	0.0222 (5)	0.0356 (6)	0.0032 (4)	0.0200 (5)	−0.0018 (5)

### Geometric parameters (Å, °)

**Table:**

O1—C1	1.3747 (12)	C8—C9	1.4181 (14)
O1—C13	1.4336 (12)	C8—H8	0.9500
O2—C14	1.2039 (13)	C9—C10	1.4354 (13)
O3—C14	1.3255 (13)	C10—C11	1.4534 (13)
O3—C15	1.4579 (13)	C11—C12	1.3438 (14)
O4—C17	1.2102 (13)	C11—H11	0.9500
O5—C17	1.3384 (12)	C12—C17	1.4763 (14)
O5—C18	1.4532 (12)	C12—C13	1.5056 (14)
C1—C10	1.3868 (14)	C13—C14	1.5376 (15)
C1—C2	1.4068 (14)	C13—H13	1.0000
C2—C3	1.3660 (15)	C15—C16	1.4978 (16)
C2—H2	0.9500	C15—H15A	0.9900
C3—C4	1.4229 (15)	C15—H15B	0.9900
C3—H3	0.9500	C16—H16A	0.9800
C4—C5	1.4176 (15)	C16—H16B	0.9800
C4—C9	1.4251 (14)	C16—H16C	0.9800
C5—C6	1.3692 (16)	C18—C19	1.5050 (16)
C5—H5	0.9500	C18—H18A	0.9900
C6—C7	1.4101 (16)	C18—H18B	0.9900
C6—H6	0.9500	C19—H19A	0.9800
C7—C8	1.3754 (15)	C19—H19B	0.9800
C7—H7	0.9500	C19—H19C	0.9800
			
C1—O1—C13	114.11 (8)	C17—C12—C13	117.40 (9)
C14—O3—C15	118.05 (9)	O1—C13—C12	110.92 (8)
C17—O5—C18	115.78 (8)	O1—C13—C14	110.70 (8)
O1—C1—C10	120.89 (9)	C12—C13—C14	112.17 (8)
O1—C1—C2	116.68 (9)	O1—C13—H13	107.6
C10—C1—C2	122.38 (9)	C12—C13—H13	107.6
C3—C2—C1	119.33 (9)	C14—C13—H13	107.6
C3—C2—H2	120.3	O2—C14—O3	126.07 (10)
C1—C2—H2	120.3	O2—C14—C13	123.27 (10)
C2—C3—C4	121.11 (10)	O3—C14—C13	110.62 (9)
C2—C3—H3	119.4	O3—C15—C16	106.18 (9)
C4—C3—H3	119.4	O3—C15—H15A	110.5
C5—C4—C3	121.31 (10)	C16—C15—H15A	110.5
C5—C4—C9	119.24 (9)	O3—C15—H15B	110.5
C3—C4—C9	119.45 (9)	C16—C15—H15B	110.5
C6—C5—C4	120.73 (10)	H15A—C15—H15B	108.7
C6—C5—H5	119.6	C15—C16—H16A	109.5
C4—C5—H5	119.6	C15—C16—H16B	109.5
C5—C6—C7	120.10 (10)	H16A—C16—H16B	109.5
C5—C6—H6	119.9	C15—C16—H16C	109.5
C7—C6—H6	119.9	H16A—C16—H16C	109.5
C8—C7—C6	120.64 (10)	H16B—C16—H16C	109.5
C8—C7—H7	119.7	O4—C17—O5	123.96 (10)
C6—C7—H7	119.7	O4—C17—C12	123.61 (9)
C7—C8—C9	120.54 (10)	O5—C17—C12	112.40 (8)
C7—C8—H8	119.7	O5—C18—C19	106.24 (9)
C9—C8—H8	119.7	O5—C18—H18A	110.5
C8—C9—C4	118.64 (9)	C19—C18—H18A	110.5
C8—C9—C10	122.48 (9)	O5—C18—H18B	110.5
C4—C9—C10	118.88 (9)	C19—C18—H18B	110.5
C1—C10—C9	118.67 (9)	H18A—C18—H18B	108.7
C1—C10—C11	117.45 (9)	C18—C19—H19A	109.5
C9—C10—C11	123.82 (9)	C18—C19—H19B	109.5
C12—C11—C10	119.55 (9)	H19A—C19—H19B	109.5
C12—C11—H11	120.2	C18—C19—H19C	109.5
C10—C11—H11	120.2	H19A—C19—H19C	109.5
C11—C12—C17	123.91 (9)	H19B—C19—H19C	109.5
C11—C12—C13	118.54 (9)		
			
C13—O1—C1—C10	−35.53 (13)	C4—C9—C10—C11	178.15 (9)
C13—O1—C1—C2	146.96 (9)	C1—C10—C11—C12	14.50 (14)
O1—C1—C2—C3	179.02 (9)	C9—C10—C11—C12	−168.35 (10)
C10—C1—C2—C3	1.55 (16)	C10—C11—C12—C17	−172.92 (9)
C1—C2—C3—C4	−2.99 (16)	C10—C11—C12—C13	2.62 (14)
C2—C3—C4—C5	−179.58 (10)	C1—O1—C13—C12	49.75 (11)
C2—C3—C4—C9	0.52 (16)	C1—O1—C13—C14	−75.44 (10)
C3—C4—C5—C6	177.24 (10)	C11—C12—C13—O1	−34.20 (13)
C9—C4—C5—C6	−2.86 (16)	C17—C12—C13—O1	141.64 (9)
C4—C5—C6—C7	−0.03 (17)	C11—C12—C13—C14	90.16 (11)
C5—C6—C7—C8	2.39 (18)	C17—C12—C13—C14	−94.01 (11)
C6—C7—C8—C9	−1.78 (17)	C15—O3—C14—O2	2.74 (16)
C7—C8—C9—C4	−1.13 (15)	C15—O3—C14—C13	−174.88 (8)
C7—C8—C9—C10	178.81 (10)	O1—C13—C14—O2	159.77 (10)
C5—C4—C9—C8	3.41 (15)	C12—C13—C14—O2	35.29 (14)
C3—C4—C9—C8	−176.69 (9)	O1—C13—C14—O3	−22.54 (11)
C5—C4—C9—C10	−176.53 (9)	C12—C13—C14—O3	−147.02 (9)
C3—C4—C9—C10	3.37 (15)	C14—O3—C15—C16	−170.34 (10)
O1—C1—C10—C9	−175.02 (9)	C18—O5—C17—O4	−4.22 (15)
C2—C1—C10—C9	2.35 (15)	C18—O5—C17—C12	174.06 (9)
O1—C1—C10—C11	2.28 (14)	C11—C12—C17—O4	170.68 (10)
C2—C1—C10—C11	179.65 (9)	C13—C12—C17—O4	−4.91 (15)
C8—C9—C10—C1	175.33 (9)	C11—C12—C17—O5	−7.61 (14)
C4—C9—C10—C1	−4.73 (15)	C13—C12—C17—O5	176.80 (9)
C8—C9—C10—C11	−1.79 (16)	C17—O5—C18—C19	173.96 (9)
